# Supplementary material for: Factors associated with pre-loss grief and preparedness in relatives of people with cancer during the COVID-19 pandemic: A cross-sectional study
Source: PLoS One. 2022 Nov 29;17(11):e0278271. doi: 10.1371/journal.pone.0278271 (PMC9707745; doi:10.1371/journal.pone.0278271)
Supplement: S1 Table — (DOCX) [file pone.0278271.s001.docx]

S1 Table. German Version of the Preparedness for Caregiving Scale.

Wir wissen, dass sich Menschen auf einige Aspekte der Pflege einer anderen Person gut vorbereitet fühlen können, während sie sich auf andere Aspekte weniger gut vorbereitet fühlen.

Wir möchten gerne wissen, wie gut Sie sich auf folgende Tätigkeiten vorbereitet fühlen, auch wenn Sie diese Art der Pflege derzeit nicht durchführen.

|  |  | Überhaupt nicht vorbe-reitet | Nicht sehr gut vorbe-reitet | Ein Stück weit vorbe-reitet | Ziemlich gut vorbe-reitet | Sehr gut vorbe-reitet |
| --- | --- | --- | --- | --- | --- | --- |
| 1 | Wie gut sind Sie Ihrer Meinung nach darauf vorbereitet, sich um die körperlichen Bedürfnisse Ihrer*Ihres Angehörigen zu kümmern? |  |  |  |  |  |
| 2 | Wie gut sind Sie Ihrer Meinung nach darauf vorbereitet, sich um seine*ihre emotionalen Bedürfnisse zu kümmern? |  |  |  |  |  |
| 3 | Wie gut sind Sie Ihrer Meinung nach darauf vorbereitet, sich über weitere Hilfen für ihn*sie zu informieren oder sie einzurichten? |  |  |  |  |  |
| 4 | Wie gut sind Sie Ihrer Meinung nach auf den Stress, den die Pflege mit sich bringt vorbereitet? |  |  |  |  |  |
| 5 | Wie gut sind Sie Ihrer Meinung nach darauf vorbereitet, Betreuungsaktivitäten für Sie und Ihre*n Angehörige*n angenehm zu gestalten? |  |  |  |  |  |
| 6 | Wie gut sind Sie Ihrer Meinung nach darauf vorbereitet, auf Notfälle zu reagieren und diese zu bewältigen, an denen er*sie beteiligt ist? |  |  |  |  |  |
| 7 | Wie gut sind Sie Ihrer Meinung nach darauf vorbereitet, die Hilfe und Informationen einzuholen, die Sie vom Gesundheitssystem benötigen? |  |  |  |  |  |
| 8 | Wie gut sind Sie insgesamt darauf vorbereitet, die Ihre*n Angehörige*n zu versorgen? |  |  |  |  |  |
