# Supplementary material for: Factors associated with pre-loss grief and preparedness in relatives of people with cancer during the COVID-19 pandemic: A cross-sectional study
Source: PLoS One. 2022 Nov 29;17(11):e0278271. doi: 10.1371/journal.pone.0278271 (PMC9707745; doi:10.1371/journal.pone.0278271)
Supplement: S2 Table — (DOCX) [file pone.0278271.s002.docx]

S2 Table. Self-generated questions for “Preparedness for death”.

|  | **If the sick person were to die soon,** |  |  |  |  |
| --- | --- | --- | --- | --- | --- |
|  |  | Not at all |  |  | Very much |
| 1 | ... would you be emotionally prepared for this? | 1 | 2 | 3 | 4 |
| 2 | ... would you already have all the information you need? | 1 | 2 | 3 | 4 |
| 3 | ... would you be prepared for it organizationally (e.g., financially)? | 1 | 2 | 3 | 4 |
