# Supplementary material for: Factors associated with pre-loss grief and preparedness in relatives of people with cancer during the COVID-19 pandemic: A cross-sectional study
Source: PLoS One. 2022 Nov 29;17(11):e0278271. doi: 10.1371/journal.pone.0278271 (PMC9707745; doi:10.1371/journal.pone.0278271)
Supplement: S3 Table — (DOCX) [file pone.0278271.s003.docx]

S3 Table. Self-generated questions for “COVID-19 related fears”.

|  | **Since the beginning of the COVID-19 pandemic, the following has changed:** |  |  |  |  |  |
| --- | --- | --- | --- | --- | --- | --- |
|  |  | Not at all |  |  |  | A lot |
| 1 | I am afraid that COVID-19 could take a severe course in the person close to me. | 1 | 2 | 3 | 4 | 5 |
| 2 | I am afraid that COVID-19 could take a severe course in me. | 1 | 2 | 3 | 4 | 5 |
| 3 | I am afraid of infecting the person close to me with COVID-19 myself. | 1 | 2 | 3 | 4 | 5 |
| 4 | I am afraid that the person close to me could be infected with COVID-19 in the course of their medical care, e.g., by medical staff or fellow patients. | 1 | 2 | 3 | 4 | 5 |
