# Supplementary material for: Factors associated with pre-loss grief and preparedness in relatives of people with cancer during the COVID-19 pandemic: A cross-sectional study
Source: PLoS One. 2022 Nov 29;17(11):e0278271. doi: 10.1371/journal.pone.0278271 (PMC9707745; doi:10.1371/journal.pone.0278271)
Supplement: S4 Table — (DOCX) [file pone.0278271.s004.docx]

S4 Table. Self-generated item for “Prognosis”.

| **How likely do you think it is that the person with cancer will die in the next 5 years?** | Not at all | Very likely |
| --- | --- | --- |
| Choose a point between 0% and 100% | 0% | 100% |
