# Supplementary material for: Factors associated with pre-loss grief and preparedness in relatives of people with cancer during the COVID-19 pandemic: A cross-sectional study
Source: PLoS One. 2022 Nov 29;17(11):e0278271. doi: 10.1371/journal.pone.0278271 (PMC9707745; doi:10.1371/journal.pone.0278271)
Supplement: S5 Table — (DOCX) [file pone.0278271.s005.docx]

S5 Table. Self-generated questions for “Health Status”.

|  | 0 | 1 |
| --- | --- | --- |
| **Do you suffer from**   - A serious chronic physical illness or physical disability OR/AND - Mental illness or disability? | No | Yes |
