# Supplementary material for: Factors associated with pre-loss grief and preparedness in relatives of people with cancer during the COVID-19 pandemic: A cross-sectional study
Source: PLoS One. 2022 Nov 29;17(11):e0278271. doi: 10.1371/journal.pone.0278271 (PMC9707745; doi:10.1371/journal.pone.0278271)
Supplement: S6 Table — (DOCX) [file pone.0278271.s006.docx]

S6 Table. Correlation matrix of all variables included in the analyses.

| Variable, *M (SD)* | Pre-loss grief,  3.49 (.77) | Preparedness for death, 5.96 (2.19) | Preparedness for caregiving, 15.10 (7.23) | Emotion-focused coping, 2.04 (.48) | Dysfunctional coping, 1.85 (.41) | Problem-focused coping, 2.52 (.59) | Depth of Relationship, 3.31 (.59) | Conflict in relationship, 1.85 (.62) | COVID-19 related fears, 12.46 (4.37) | Attachment avoidance, 2.97 (1.79) | Attachment anxiety, 2.49 (1.42) | Age, 41.35 (12.21) | Prognosis, 59.77 (37.46) |
| --- | --- | --- | --- | --- | --- | --- | --- | --- | --- | --- | --- | --- | --- |
| Pre-loss grief |  | -.322*** | -.181** | -.387*** | .350*** | -.102 | .409*** | -.083 | .226*** | .098 | -.008 | -.138* | .258*** |
| Preparedness for death | -.322*** |  | .446*** | .200*** | -.215*** | .124* | -.196*** | -.004 | -.133* | -.037 | -.105 | .322*** | .312*** |
| Preparedness for caregving | -.181** | .446*** |  | .208*** | -.097 | .227*** | .110 | -.200*** | -.114 | -.036 | -.255*** | .148* | .013 |
| Emotion-focused coping | -.387*** | .200*** | .208*** |  | .036 | .473*** | .010 | .039 | -.057 | -.142* | -.104 | .003 | -.214*** |
| Dysfunctional coping | .350*** | -.215*** | -.097 | .036 |  | .167** | .231*** | .092 | -.025 | .052 | .069 | -.195*** | .021 |
| Problem-focused coping | -.102 | .124* | .227*** | .473*** | .167** |  | .159** | -.009 | -.017 | -.153** | -.104 | .015 | -.064 |
| Depth of Relationship | .409*** | -.196*** | .110 | .010 | .231*** | .159** |  | -.221*** | .202*** | -.130* | -.112 | -.116* | -.153** |
| Conflict in Relationship | -.083 | -.004 | -.200*** | .039 | .092 | -.009 | -.221*** |  | -.117* | .225*** | .251*** | .229*** | .003 |
| COVID-19 related fears | .226*** | -.133* | -.114 | -.057 | -.025 | -.017 | .202*** | -.117* |  | .030 | .048 | -.113 | -.002 |
| Attachment avoidance | .098 | -.037 | -.036 | -.142* | .052 | -.153** | -.130* | .225*** | .030 |  | .198*** | .098 | .002 |
| Attachment anxiety | -.008 | -.105 | -.255*** | -.104 | .069 | -.104 | -.112 | .251*** | .048 | .198*** |  | -.052 | -.039 |
| Age | -.138* | .322*** | .148* | .003 | -.195*** | .015 | -.116* | .229*** | -.113 | .098 | -.052 |  | .114* |
| Prognosis | .258*** | .312*** | .013 | -.214*** | .021 | -.064 | -.153** | .003 | -.002 | .002 | -.039 | .114* |  |

Note. * p<.05; ** p<.01; *** p<.001
