# Supplementary material for: Factors associated with pre-loss grief and preparedness in relatives of people with cancer during the COVID-19 pandemic: A cross-sectional study
Source: PLoS One. 2022 Nov 29;17(11):e0278271. doi: 10.1371/journal.pone.0278271 (PMC9707745; doi:10.1371/journal.pone.0278271)
Supplement: Attachment — Submitted filename: PONE-D-22-17257_reviewer Comments.docx [file pone.0278271.s007.docx]

PONE-D-22-17257
Factors associated with pre-loss grief and preparedness in relatives of people with cancer during the COVID-19 pandemic: A cross-sectional study.

**Reviewer comments**

Line 50. Include the reference for first sentence.

Line 61-68. The paragraph contains in-text citations with page numbers, I am not sure if that conforms with PLOS ONE guidelines.

Line 71. ….. were a risk factor (for what?)

Page 125-132 H1-H3 The hypotheses should be in future tenses. …will be associated… not ….is associated……

Page 133-134 Furthermore, we assume that quality of relationship to the patient and prognosis of the patient are (use future tense, will be) associated

Page 146-7 ..social networks? You mean social media networks?

Page 152-153 Why the use of convenient sampling?

Page 158-159 Were the participants able to submit the survey after completing only demographic information? How dropouts were obtained is not clearly stated

Page 159-160 How many times were the survey(s) completed? You reported additional dropouts during the remaining online surveys

Page 162 Was written informed consent really provided? I thought they indicated their consent in the online survey link. Maybe you rewrite?

164 Measures sub heading. For all the scales, did you use the German versions or you translated them? Give details. Also, for Table 1, is it possible to indicate which scores indicate better outcomes for the rest of the scales? You singled out a few in the procedure and mention higher scores indicates………

Page 233…… while the opposite was true…… the statement can confuse the reader in interpreting the result. Use straightforward language

Page 235 Table 3. Is it possible to indicate p-values that are significant? It makes reading through easy

Page 261 under discussion. The sentence that starts with ‘In accordance with our first hypothesis…..’ needs corrections

Page 267 Regarding our second hypothesis (not hypotheses)

Page 309 under limitations. It will be good to discuss how that women dominance in the sample might have affected the result. Are there studies indicating which gender is associated with more grief and other outcomes of concern in this study? The same goes to literacy level. Majority of the participants are highly educated. Any literature on literacy level and the outcomes of this study?

Did you collect health information of the participants themselves? Don’t you think their health status may also affect their responses?

Another potential limitation, did you ask how much information the participants know about the cancer patients? The amount of knowledge they have will definitely play a role in their grief and preparedness
