# Supplementary material for: Factors associated with pre-loss grief and preparedness in relatives of people with cancer during the COVID-19 pandemic: A cross-sectional study
Source: PLoS One. 2022 Nov 29;17(11):e0278271. doi: 10.1371/journal.pone.0278271 (PMC9707745; doi:10.1371/journal.pone.0278271)
Supplement: Attachment — Submitted filename: Reviewer comment cancer and Covid grief.docx [file pone.0278271.s008.docx]

**Abstract.**

Objectives: Please be clear on what you mean by constructs in the following phase ‘and their relationship with COVID-19 specific constructs.’ Remember the abstract is the gateway to your article.

Method: It should be ‘Data of 299 participants from a cross-sectional study was used.

Please be very clear about the analysis. What did you use Multivariate linear regression analysis for? What were the independent and the dependent variables?

From what I understand the dependent variables are Pre-loss grief, Preparedness for death, and Preparedness for caregiving; while the independent variables are perceived depth of the relationship, COVID-19 related fears, prognosis for death, dysfunctional coping strategies, emotion-focused coping strategies, prognosis for death, problem-focused coping, attachment anxiety and perceived conflict in the relationship with the cancer patient. So if I am right, in reporting your result, you should say the independent variable (name) significantly or not predicted the dependent variable (name).

Also please use patients with cancer in place of cancer patients

I think it will be good if you can include r values and the 95% CI together with the β and *p* values. This will make the result more meaningful

Conclusion: It should be ‘COVID-19 pandemic impacts on the grieving process of relatives of patients with cancer. Consequently, screening for pre-loss grief, preparedness and their associated factors may help provide early support for relatives of people with cancer at need. However, further research is needed to help understand the stability of pre-loss grief and preparedness.

Introduction

Although the introduction has provided many relevant information, it is way too long, and it has included many irrelevant information. I think it will be good if the authors will talk about cancer in one paragraph and how death is expected in the patients, and then connect this with pre-loss grief and preparedness in the relatives of the patients in the second paragraph. The third paragraph can talk about the problem and the aim of the study. This way, the introduction will be more succinct.

Procedure

Line 144: It should be ‘and was approved’

Page 7

Please take the following to the result section: In total, 646 potential participants started the online survey. Of these, 273 dropped out during or after completing the sociodemographic variables. Further 74 participants were excluded due to dropping out during the remaining online survey, leading to a total number of *n*=299 participants.

What you need under this section: are inclusion and exclusion criteria, the characteristics of the participants such as being a relative of patients with cancer, participants who can speak German, etc, etc.

Measures

I do not know why you need Table 1? What we need to see under measures are the description, characteristics and the psychometric properties of the outcome measures from previous studies, unless they are new measures invented by you.

Statistical analysis

Because three different models were tested and to correct for Type 1 error, we applied Bonferroni correction and set the significance level at p<.0167. I am not sure Bonferoni correction was appropriately applied. Bonferonni correction is used when there is multiple pairwise comparison, and in the first, you are not comparing anything with anything.

Table 3: You need to provide r values (rank order correlation) in the table. The 95% CI is for the r value.

Also please	cross-check the p values in the text. Some are reading p<000

**Factors associated with preparedness for caregiving**

You mentioned power analysis here: A power analysis with G*Power 3.1. revealed a medium sized effect (f2 = .22, α = .0167, power = .80). What is it for?

Limitation

This is not limitation: Furthermore, the cross-sectional design and exploratory analyses may limit the generalizability of results.

Please take of the limitation to the discussion section. They are not limitations.

Conclusion: The conclusion is too long. A conclusion should be very succinct.
